# The study on the impact of short video tourism Vloggers at social media platform on online sharing intention

**DOI:** 10.3389/fpsyg.2022.905002

**Published:** 2022-07-26

**Authors:** Chen Zhao, Huawen Shen, Yating Zhang

**Affiliations:** ^1^International School of Cultural Tourism, Zhejiang University City College, Hangzhou, China; ^2^Faculty of International Tourism and Management, City University of Macau, Macau, China

**Keywords:** short video Vloggers, emotional engagement, value congruence, entertainment motivation, parasocial intention, sharing intention

## Abstract

COVID-19 has caused significant damage globally, including tourism. This study adopts the quantitative research method, selects 588 samples from tourists watching short videos to investigate the antecedents and effects of parasocial interaction between tourists and short video tourism Vloggers, and analyses them with partial least squares. Based on parasocial relationship theory, this study investigates the antecedents of parasocial relationships between tourists and short video tourism Vloggers and their willingness to share short video tourism. Results show that the consistency of values, entertainment motivation, and emotional engagement positively impact the parasocial relationships between tourists and short video tourism Vloggers and affect the online sharing intention through the parasocial relationship. The consistency of values can directly affect sharing intention. As an intermediary variable, parasocial relationship positively impacts value congruence, entertainment motivation, emotional engagement, and sharing intention. This study introduces parasocial relationship into the research of tourism short video Vloggers, which enriches the literature. Furthermore, this introduction provides new marketing strategies and suggestions for the sustainable development of tourism.

## Introduction

As one of the most powerful online network tools, social media has been integrated into the social and economic life of the real world. Short videos are a new force suddenly gaining popularity. With the development of the Internet economy, XI GUA, TikTok, and other short video platforms have attracted many short video practitioners and audiences. According to the 46th statistical report on China’s Internet Development released by China Internet Network Information Center in Beijing on September 29, 2020, the number of users of short videos reached 873 million, accounting for 88.3% of the total number of Internet users (CNNIC, 2020). Short videos have a wide range of applications, especially in the tourism industry. As a mainstream form of social media, the information in short travel videos can guide visitors in their choice of destination. According to the research report on the demand trend of China’s leisure tourism customers, jointly released by China Tourism Research Institute and Ctrip Travel Network, more than 40% of domestic tourists have used websites, BBS, or forums to obtain travel information, and The proportion of inbound visitors using online media is over 60% and the proportion of outbound visitors is over 50% (Net, 2020). In tourism, social media directly affects tourists’ decision-making and changes their travel behavior (Xiang and Gretzel, 2010; Hudson and Thal, 2013). The most intuitive effect of social media in tourism is to increase the number of visits to destinations, which is beneficial to the sustainable development of tourism destination brands (Chen and Zhang, 2015). As a virtual platform for tourists to share their travel experiences and emotions, short videos have gradually become the reference basis for potential tourists to make travel decisions (Chen et al., 2013). Short video tourism Vloggers are content creators in online tourism who display tourism experiences and provide tourism suggestions through video-generated content no longer than 5 min, establishing an emotional connection with their followers through interactive communication (Gao, 2018). Videos bring new opportunities to tourism destinations (Hu and Guo, 2020). Using social media to market tourism has proved to be a good strategy (Fotis et al., 2012). Scholars (Tung and Ritchie, 2011; Munar and Jacobsen, 2014) believe that social media has fundamentally changed personal travel planning.

In reviewing previous studies on social media and tourism, most of them are based on hotel websites (McCarthy et al., 2010; Sparks and Browning, 2011), tourism websites (Milano et al., 2011), and blogs (Pan et al., 2007; Akehurst, 2009; Leung et al., 2011) on the types of social media, such as WeChat (Liang and Yang, 2018; Xiao, 2019; Jiang et al., 2020). For the sustainable development of tourism, sharing tourism information is also an important factor. Sotiriadis (2017) reviewed articles sharing travel experiences on social media. According to the literature on sharing intention, most are based on information content (Yang et al., 2017), emotion (Wang et al., 2017), attitude (Lee et al., 2016), perceived usefulness (Ma et al., 2018), EWOM (Jalilvand et al., 2012) and user group (Zhao et al., 2020). Su et al. (2021) supposed Tourism activity type is the key factor leading to different sharing content and Tourist well-being is an important mechanism for travel experience sharing. However, short videos have not been thoroughly studied as a sustainable online communication channel considering its current characteristics and the imagined intimate relationship through parasocial relationship. Furthermore, what attracts visitors to interact with tourism Vloggers and how this interaction leads to the promotion of tourists’ sharing intention are still unclear. In the context of parasocial relationships, the influencing factors of short video tourism Vloggers’ online sharing intention are a new direction. Based on the sustainable development of tourism, this study uses parasocial relationship theory and similarity attraction theory to fill the above research gap.

In other words, this study determines the role of short video tourism Vloggers, emotional engagement, value congruence, parasocial relations and entertainment motivation in influencing sharing intention. The following objectives guide this study: to explore the impact of emotional engagement, value congruence and entertainment motivation on parasocial relationships and examine the impact of parasocial relations on sharing intention and the intermediary role of parasocial relations.

This study has particular theoretical significance. Firstly, parasocial relationship theories in social media are based on websites or public accounts. This study takes short video tourism Vloggers on the social media platform as the research object, expanding the parasocial relationship theory application scope. Second, short video tourism blogging is a new research direction, which expands the factors of tourism sustainable development. This study expands the existing parasocial relationship literature by revealing the antecedents and consequences of parasocial relationship and online sharing intention. Finally, this study increases the existing parasocial relationship literature by revealing the antecedents and consequences of parasocial relations and online sharing intention.

## Literature review

### Emotion engagement and parasocial relationship

The definition of emotional engagement varies among different research backgrounds. Emotional engagement is mainly used in the field of research and learning. Marks (2000) believes that emotional engagement is a psychological state in which much energy and attention are spent to complete a learning task. Fredricks et al. (2004) define emotional engagement as “a person’s emotional reaction when undertaking a specific task,” which ultimately includes “positive and negative reactions to teachers, classmates, scholars and schools, and is considered to establish contact with an institution and affect work Willingness.” Hilvert-Bruce et al. (2018) and Guo (2018) believe that emotional engagement refers to emotional connections and expressions of emotions of audience of live programs through responses to performers and other audience. Viewers interact with streaming media and other viewers through instant chat to obtain an alternative experience (Lim et al., 2020). Emotional engagement means that tourists can feel the emotional connection with short video tourism Vloggers. The feeling of emotional connection comes from the nature of fast-moving instant chat with other users, who will respond to each other’s comments and questions, some of which include serious comments on streaming media. When the audience is in a fast-moving instant chat environment, they may experience the phenomenon of immersion or a “spiritual feeling of devotion,” which promotes them to contact with others actively and participate emotionally; emotional connectivity (Guo, 2018; Hilvert-Bruce et al., 2018) and emotional expression (Lim et al., 2015) can lead to parasocial relationships. Researchers believe that the audience’s ability to understand the emotional response of others is closely related to parasocial relationships (Davis et al., 1987). Kim (2012) support that Emotional involvement is a main driver affecting film tourism experiences. Watching short videos may have a positive relationship with tourists. Thus, if users feel more emotional contact with their short video travel Vloggers, they will have a stronger sense of parasocial relationships. Previous studies by scholars emphasized the importance of emotional participation in changing people’s behavior (Ramkissoon et al., 2013; Dewnarain et al., 2019; Majeed and Ramkissoon, 2022). Wang et al. (2017) explored the influencing factors of positive emotion on online sharing hospitals on microblogs. An online questionnaire survey was conducted among 341 microblog users. The results show that positive emotion has a positive impact on sharing intention. An increase of tourists’ emotion may lead to the behavior or intention of sharing information to others. Based on previous studies, this study puts forward the following hypotheses:

*H1*: Tourists’ emotional engagement has a positive impact on social interaction.

*H2*: Tourists’ emotional engagement has a positive impact on sharing intention.

### Entertainment motivation and parasocial relationship

People have the opportunity to communicate through potential motivation (Rubin and Step, 2000), and the motivation of social media users to use media may affect parasocial relationship (Rubin et al., 1985). Similarly, tourists using social platforms to watch tourism Vloggers also have the opportunity to generate parasocial relations. While watching short videos, people have a certain sense of entertainment (Haridakis and Hanson, 2009). Kawamura et al. (2009) believe that entertainment will promote the development of parasocial relationships because viewers prefer to pay attention to information that can meet their motivation (Rubin and Step, 2000). Viewers with entertainment motivation will pay more attention to the entertainment value of video (Sokolova and Kefi, 2020), which makes them feel closer to short video tourism Vloggers. Based on previous studies, this study puts forward the following hypothesis:

*H3*: The entertainment motivation of tourists and short video tourism Vloggers positively impacts sharing intention.

### Value congruence and parasocial relationship

Value congruence originates from the fit theory between humans and the environment (Audia et al., 1996). It describes how the environment meets human needs, values or references. Complementary adaptation occurs when a person “complements, modifies or has characteristics similar to those of other individuals in the environment” (Muchinsky and Monahan, 1987). The concept of value congruence first appeared in academia when it was applied to the study of organizational behavior and employee relations (Zucker, 1987). Later, in marketing, it refers to the personal values of consumers and the values they believe exist in the organization (Zhao et al., 2012). The degree of congruence between the main value of the video and the tourists in this study refers to the congruence of their values in tourism. Concerning the use of social media, Ayeh et al. (2013) found that user-generated content (UGC) contributors and focused customers affect perceived source credibility and subsequent attitudes and intentions to use UGC. The similarity between value congruence and social media speakers may lead to customers’ positive attitudes and behavioral intentions. The attitude similarity between TV performers and viewers promotes parasocial relationships. Thus, we believe that tourists are likely to form a positive attitude and even have a sense of intimacy similar to that of short video travel Vloggers, who are considered to have personality. On the basis of the same values, customers are highly likely to have a good impression on short video tourism Vloggers and find their tourism information credible. Thus, customers will engage in collecting, forwarding, liking and other behaviors. Therefore, value congruence can change people’s behavior intention to a certain extent.

Based on previous studies, this study puts forward the hypotheses:

*H4*: Value congruence between the tourist and short video tourism Vloggers positively impacts parasocial relationships.

*H5*: Value congruence between tourists and short video tourism Vloggers positively impacts sharing intention.

### Parasocial relationship and sharing intention

Sharing intention is defined as the intention of online users to find the information useful to others, attract others’ attention on the Internet and share this information with others (Erdelez and Rioux, 2000). Horton and Richard Wohl (1956) studied the performance and response of performers and audience in TV programs. It showed that an intimate relationship was established between performers and audience through this interaction. It also discussed how this “intimacy” was established through examples. In this study, parasocial relationships refer to imaginary intimate relationships, which is a sense of intimacy. Many parasocial relationships have been used to study the phenomenon of star chasing. The stronger their parasocial relationship with stars, the higher their positive emotions (Li, 2015). Previous studies have shown that the stronger the emotion aroused, the stronger the feeling of interaction, and the greater the possibility of sharing or sharing intention (Berger and Milkman, 2010; Berger, 2011; Nelson-Field et al., 2013; Hagerstrom et al., 2014). Based on previous studies, this study puts forward the following hypothesis:

*H6*: The parasocial relationship between tourists and short video tourism Vloggers positively impacts online sharing intention.

### Mediating effect of parasocial relationships

**Figure 1:**
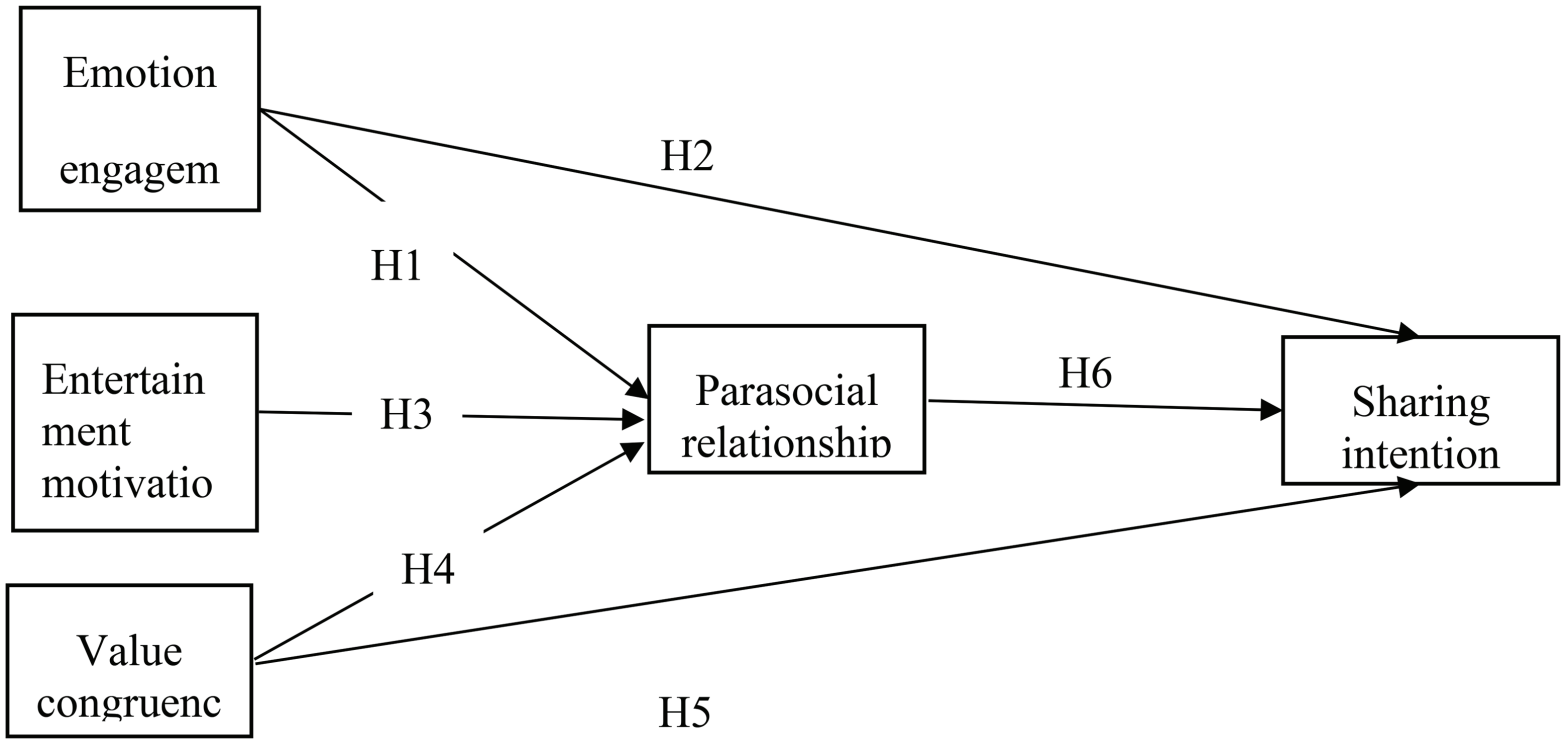
Proposed research model.

Parasocial relationships are an important factor connecting the relationship between people. Parasocial social interaction in social media mostly refers to imaginary intimate relationships (Haobin Ye et al., 2021). Many studies have shown that quasi-social interaction can affect people’s behavior or attitude. In the study of social media, parasocial relationships have an intermediary effect. Zheng et al. (2020) in the research on the role of attraction and parasocial relationship in social shopping websites, using three variables, namely, physical attraction, social attraction and technology attraction, and using technology attraction theory and parasocial relationship theory, this study analyses how the three types of technology attraction affect parasocial relationships to affect users’ social business intention. Ramkissoon and Mavondo (2015) supportive emotions can affect behavioral intention. Liu et al. (2019), in the research on virtual blogging and brand evaluation, the impact of parasocial interaction, through a short video survey, authors are interested in understanding which video blog (vlogger) can better help their marketers develop their brand image. In contrast, vlog viewers tend to evaluate the positive brand recognized by vloggers and how these effects occur. The results show that parasocial relationship has a complete mediating effect on the impact of physical attractiveness on perceived brand. In tourism, in the study of tourism websites, parasocial relationship plays an intermediary role in value consistency, perceived consistency and civic behavior. The above research shows that parasocial relationship has the possibility of mediation effect (Figure 1). Based on previous studies, this study puts forward the following hypothesis:

*H7*: Parasocial relations have a mediating effect.

## Research methods

### Research context

This study uses quantitative research methods to take tourists who watch short video tourism Vloggers as the object. Most tourists prefer obtaining information through short video tourism Vloggers because it has the characteristics of vividness, interaction and authenticity. This study discusses the relationship between tourists watching short video tourism Vloggers and their willingness to share from the aspects of value congruence, entertainment motivation, emotion engagement and parasocial interaction.

### Questionnaire development and measurements

A questionnaire was developed to obtain quantitative data. The questionnaire is divided into three parts. The first part includes screening questions to identify qualified participants for the current study. The respondents in this study are tourists who have watched short video tourism Vloggers’ information in the past 6 months. The second part is 25 seven-point Likert scale questions, which evaluate the five structures of the proposed framework: value congruence, entertainment motivation, parasocial relationship, emotional engagement and sharing intention.

The study conducted a questionnaire survey using the existing measurement scales in the early study. Entertainment motivation was measured by items from Sokolova and Kefi (2020; e.g., “I watch video blog because it is entertainment”). Value congruence was measured by three items from Jung and Avolio (2000; e.g., “I have a clear understanding of what the core values of the influencer mean”). Parasocial relationships are measured using eight items from Rubin et al. (1985) and Kim et al. (2015; e.g., “I would like to meet the influencer in person”). Four items developed by Lim et al. (2015) and Lim et al. (2020) measured emotional engagement (e.g., “I quoted the live streamer or commentator when influencer said something good or witty”). Finally, willingness to share online is measured by three projects developed by Wang et al. (2017; e.g., “I have a strong desire to share the short-sightedness of the tour Frequency”). Previous studies have confirmed that all scales used in this study are reliable and effective. Preston and Colman (2000) and Lietz (2010) believe that the seven-point Likert scale is more reliable and differentiated than the five-point scale, and it is also the best way of data skew distribution (Bollen, 1989). All items are scored on a seven-point scale from “strongly disagree” (1) to “strongly agree” (7). The third part is the demographic characteristics of the subjects, including gender, age, education, marital status, occupation and income.

The questionnaire was originally designed in English. The questionnaire was translated into Chinese through reverse translation technology to facilitate the distribution of the questionnaire to online respondents. The Chinese and English versions were sent to English professors to verify the accuracy of content expression. To evaluate the validity of the content, a pilot test of 120 participants was conducted in October 2021 to confirm the content further, and the wording was slightly modified.

### Sampling, data collection and analysis

Given the inability to grasp the information of those who watch short clips of tourism Vloggers’ videos, this study used the convenient sampling method to issue the questionnaire. we were distributed the survey questionnaire online and the data was collected it from Nov.1 to Dec.28, 2021, on WEN JUAN XIONG^1^ platform. To achieve the purpose of pre-investigation, the researcher asked questions before the investigation volume evaluation. Participants were 10 audiences who often used social media to watch short video travel blogger information. They were mainly asked to evaluate the reliability of five main concepts and modify the confusing description in the expression.

In terms of total sample size, Hair et al. (2011) pointed out that, for structural equation modeling, the sample size should exceed 10 times the number of estimated variables to produce reliable results. Considering that the questionnaire of Items is 25, the sample size of this survey is 588. Of the 588 questionnaires initially received, 46 were invalid. For 542 available answers, the effective rate was 92%.

This study uses the partial least squares SEM (PLS-SEM) analysis method for CFA and path analysis. PLS-SEM has significant advantages in dealing with complex models and exploratory research models (Hair et al., 2011). PLS-SEM adopts the square of step-by-step estimation parameters, covariance-based SEM and PLS-SEM. PLS-SEM calculation generally includes two stages. In the first stage, the score of the construct is estimated. In the second stage, the factor loads/weights of the measurement model and the path coefficients of the path model are calculated. Through descriptive analysis, the demographic characteristics of respondents and descriptive information of all variables are obtained. Composite reliability (CR) and ρ check the internal reliability of the structure. Convergence effectiveness and discriminant effectiveness are used to test the effectiveness of all structures. After testing the measurement model, SEM was used to test the hypothesis.

## Results

### Respondent demographics

In terms of gender, women account for 54.2%, and men account for 45.8%. Women are slightly more than men, but the two are balanced, reflecting the characteristics of the Internet. Young people account for more than the elderly. In terms of age distribution, subjects aged 18 to 25 account for the largest proportion, accounting for 62.4% of the whole sample, and subjects aged over 60 account for at least 1%, reflecting the characteristics of the Internet. Young people account for more than the elderly. In terms of education level, most of the population has received higher education, with undergraduate accounting for the most, accounting for 44.8% of the overall proportion, and 0.6% below the primary school. From this point of view, the audience of short video tourism Vloggers are mostly young people, especially students. From this point of view, the audience of short video tourism Vloggers is mostly young people, especially students. Regarding the nature of work, the proportion of bachelor’s degree is the most, accounting for 39.9% of the overall proportion, and the proportion of retirees is the least, accounting for 0.4% of the overall proportion. These proportions show that the audience of short video tourism Vloggers are people who are relatively free in time. From the perspective of attention time, 144 people have been paying attention for more than 3 years, accounting for the highest proportion of 26.6 and 16.1% within 1 year, indicating that the audience watching short videos are long-term users and have a certain stability. From the perspective of personal monthly income, most of the audience are concentrated between 2000 and 8,000, proving that the audience can travel to a large extent (Table 1).

**Table 1:** Respondent profiles (*n* = 542).

	Category	Frequency	%
Gender	Male	248	45.8
	Female	294	54.2
Age	18–25	338	62.4
	26–30	129	23.8
	31–40	60	11.1
	41–50	13	2.4
	51–60	1	0.2
	Above 60	1	0.2
Education	High school degree or below	80	14.8
	College Diploma	150	27.7
	Bachelor’s degree	243	44.8
	Master’s degree or above	69	12.7
Personal Annual Income (Unit: RMB)	2,000 or below	160	29.5
	2,001–4,000	118	21.8
	4,001–6,000	91	16.8
	6,001–8,000	75	13.8
	8,001–10,000	47	8.7
	Above 10,000	51	9.4
Occupation	Student	216	39.9
	Private owners	30	5.5
	Enterprise staff	179	33
	retiree	2	0.4
	Freelancer	72	13.3
	Others	43	7.9
Focus on time	6 months or below	97	17.9
	1 year below	87	16
	2 years below	110	20.3
	2–3 years	104	19.2
	Above 3 years	144	26.6

### Measurement model

Harman’s single factor score examines the variance of common methods to determine any potential deviation caused by the measurement method (Podsakoff et al., 2012). The total variance explained by the single factor was 43.67% for the sample, which was below the cut-off point of 50%, Indicates that there are no serious common method biases.

Table 2 shows that the ρAs values ranged from 0.817 to 0.904 and the CR values ranged from 0.879 to 0.929. The threshold of ρAs value is above 0.7 (Cronbach, 1951). The value of CR ranged from 0.7 to 0.95. Therefore, the internal consistency reliability of the measurement model can be confirmed. Evaluating the factor loading and extracted average variance (AVE) can show whether the convergent validity is up to standard. The threshold of factor loading is above 0.7 (Chin and Newsted, 1999), and the factor loadings of all items are higher than 0.7 (0.701–0.888), indicates good reliability and validity. As shown in Table 2, all AVE values ranged from 0.584 to 0.814, which are above the threshold of 0.5, showing a good convergent validity for this model.

**Table 2:** Results of confirmatory factor analysis.

Factor	Item	Standardized estimate	ρAs	Composite reliability	Average variance extracted (AVE)
Entertainment motivation	I watch short video tourism Vloggers to fill my free time	0.746	0.860	0.894	0.584
	I watch short video tourism Vloggers because it is entertaining	0.797			
	I watch short video tourism Vloggers pass time when I am bored	0.794			
	I watch short video tourism Vloggers because it is relaxing	0.708			
	I watch short video tourism Vloggers because it is cool to watch it	0.779			
	I am excited when I watch short video tourism Vloggers	0.758			
Value congruence	I really support the intent of the core values of the short video tourism Vloggers	0.888	0.847	0.907	0.766
	I agree with the core values of the short video tourism Vloggers	0.881			
	I have a clear understanding of what the core values of the short video tourism Vloggers mean	0.811			
Parasocial relationship	I look forward to watching the short video tourism Vloggers	0.789	0.904	0.921	0.593
	If the I look forward to watching the short video tourism Vloggers in short video appeared on another channel, I would watch	0.762			
	When I am watching the, I feel as if I am part of her group	0.776			
	I would like to meet the short video tourism Vloggers in person	0.755			
	If there were a story about the short video tourism Vloggers in a newspaper or magazine, I would read it	0.710			
	The short video tourism Vloggers makes me feel comfortable, as if I am with a friend	0.780			
	I find the short video tourism Vloggers attractive	0.822			
	Visiting the short video tourism Vloggers social media site makes me relax	0.762			
Emotion engagement	I quoted the live-streamer or commentator when short video tourism Vloggers said something good or witty	0.792	0.817	0.879	0.645
	I expressed my feelings about the short video tourism Vloggers or commentators in chats	0.834			
	sometimes use an emote when short video tourism Vloggers said something good or witty	0.806			
	When I participate in short video tourism Vloggers chat, I feel emotionally connected with users I am chatting with	0.780			
Sharing intention	I have had the urge to share short travel videos many times	0.902	0.887	0.929	0.814
	I have a strong desire to share this short travel video	0.912			
	I suddenly want to share this short travel video	0.892			

Discriminant validity was tested using two approaches: Fornell–Larcker criterion analysis and the Heterotrait–Monotrait Ratio of Correlations (HTMT). Table 3 shows that the square roots of AVEs on each construct are greater than the correlations between constructs (Fornell and Larcker, 1981; Net, 2020). HTMT ratios, as shown in Table 4, were all lower than 0.85 (Henseler et al., 2015). These two approaches showed that satisfactory discriminant validity was established. Thus, the convergent validity of the measurement model can be confirmed.

**Table 3:** Latent variable correlation coefficients.

	Emotion engagement	Entertainment motivation	Parasocial relationship	Sharing intention	Value congruence
Emotion engagement	0.803				
Entertainment motivation	0.660	0.764			
Parasocial relationship	0.684	0.745	0.770		
Sharing intention	0.359	0.359	0.445	0.902	
Value congruence	0.555	0.618	0.606	0.344	0.875

**Table 4:** Heterotrait–Monotrait Ratio of Correlations (HTMT) analysis.

	Emotion engagement	Entertainment motivation	Parasocial relationship	Sharing intention	Value congruence
Emotion engagement					
Entertainment motivation	0.784				
Parasocial relationship	0.794	0.842			
Sharing intention	0.422	0.409	0.485		
Value congruence	0.667	0.723	0.692	0.396	

### Structure model

A total of 542 samples were used in this study to test the research model. The bootstrapping sample size is 5,000 to evaluate the statistical significance of entertainment motivation, value congruence, emotion engagement, parasocial relationship and sharing intention, with a 95% confidence interval. Test collinearity, test of the significance of path coefficients and examination of the level of coefficients of determination or R2 evaluation to model for evaluation. The variance inflation factor (VIF) was used to test for collinearity (Hair et al., 2016). The study results show that all VIFs are below 5, ranging from 1.542 to 2.516, suggesting that multicollinearity was not an issue in this study (Hair et al., 2011).

Table 5 reports the estimated path coefficients in the research model. Specifically, emotion engagement (*β* = 0.297, *t* = 9.772, *p* = 0.000), entertainment motivation (*β* = 0.297, *t* = 9.772, p = 0.000) and value congruence (*β* = 0.166, *t* = 5.566, *p* = 0.000) have positive effects on parasocial relationship. Parasocial relationship (*β* = 0.337, *t* = 2.207, *p* = 0.000) and value congruence (*β* = 0.119, *t* = 19.143, *p* = 0.027) have a positive effect on Sharing intention. Lastly, emotion engagement (*β* = 0.034, *t* = 6.898, *p* = 0.617) to sharing intention has no impact.

**Table 5:** Path coefficients in the structural model.

Path	*β*	*t*	*p*	Support
H1 Parasocial relationship←Emotion Engagement	0.296	6.224	0.000	Yes
H2 Sharing intention←Emotion Engagement	0.089	1.482	0.138	No
H3 Parasocial relationship←Entertainment motivation	0.448	9.234	0.000	Yes
H4 Parasocial relationship←Value congruence	0.166	4.099	0.000	Yes
H5 Sharing intention←Value congruence	0.119	19.143	0.027	Yes
H6 Sharing intention←Parasocial relationship	0.108	2.014	0.000	Yes

**Figure 2:**
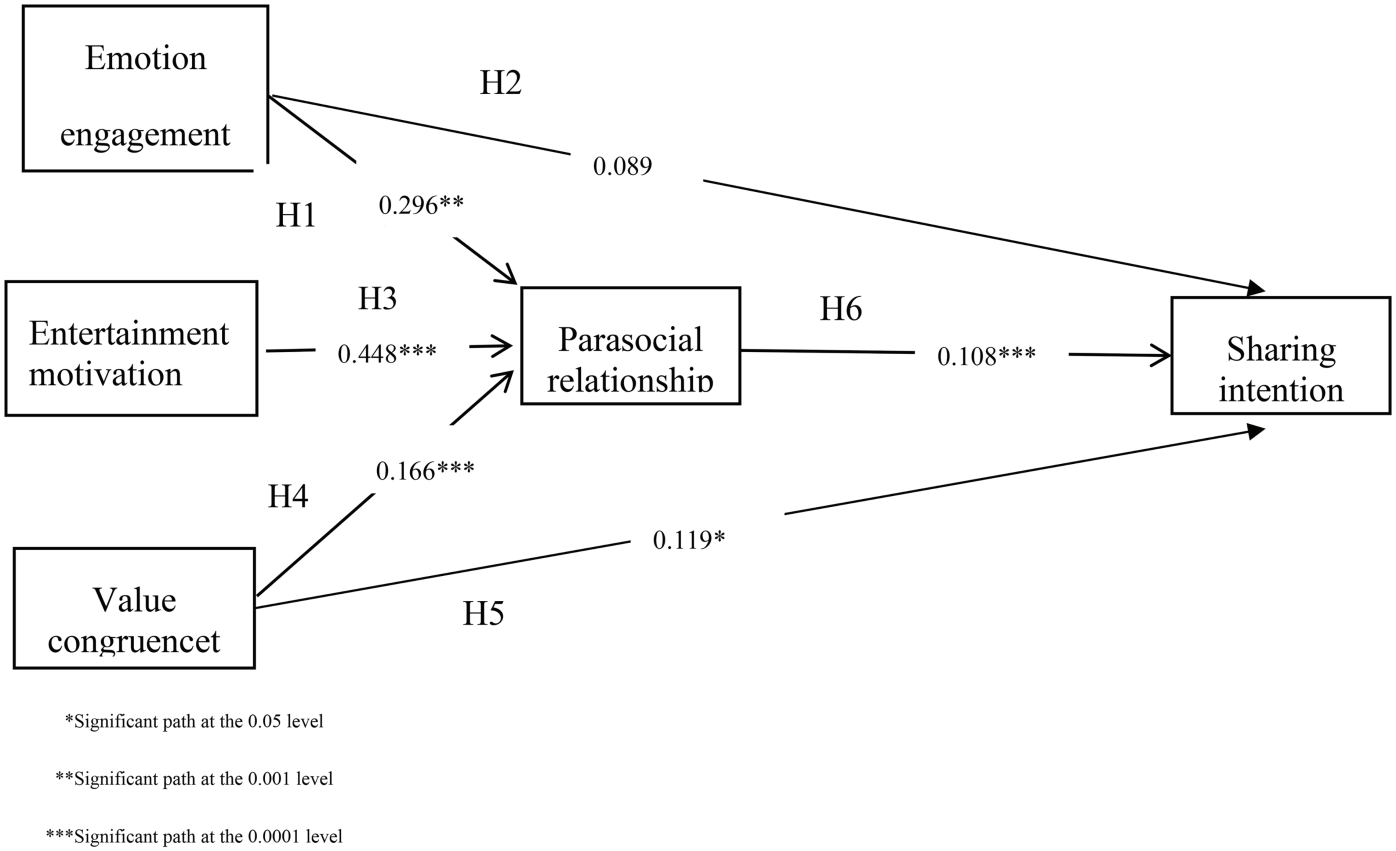
Final structural model with standardized path coefficients.

The bootstrapping resampling method was used to test the mediation role of emotional engagement. The interval is less than 0.05, which supports the mediation effect. In Table 6, parasocial relationship mediates the relationship between value congruence and sharing intention (*β* = 0.056, *t* = 3.126, *p* = 0.002); parasocial relationship mediates the relationship between entertainment motivation and sharing intention (*β* = 0.100, *t* = 3.605, *p* = 0.000), and parasocial relationship mediates the relationship between emotion engagement and sharing intention (*β* = 0.151, *t* = 4.327, *p* = 0.000). Therefore, all the hypotheses H1, H3, H4, H5 and H6 were supported, and H2 was not supported. Figure 2 shows the research model with all proposed relationship and their results. The determination coefficient (R^2^) is used to measure prediction accuracy and represents the overall effect of all external variables on internal dependent variables. The R^2^ values of 0.75, 0.50 and 0.200 can be classified as significant, moderate and weak explanatory power (Henseler et al., 2009; Hair et al., 2011). In Table 7, all R^2^ values in this model sit between 0.203 and 0.636 (parasocial relationship R^2^ = 0.636 and sharing intention R^2^ = 0.200). Therefore, the results of the R^2^ value in this study are satisfactory. These results indicated that the model had satisfactory prediction accuracy. Predictive relevance (Q^2^) larger than zero indicates that the prospective variable has predictive relevance for a certain dimension (Chin, 1998; Henseler et al., 2009). Table 7 also reports that all of the Q^2^ assessment results are larger than zero, indicating that the structural model in this study has adequate predictive capacity.

**Table 6:** Specific indirect effects in the structural model.

Path	*β*	*t*	*p*	Support
Value congruence→Parasocial relationship→Sharing intention	0.056	3.126	0.002	Yes
Entertainment motivation→Parasocial relationship→Sharing intention	0.100	3.605	0.000	Yes
Emotion Engagement→Parasocial relationship→Sharing intention	0.151	4.327	0.000	Yes

**Table 7:** R^2^ and Q^2^.

	R^2^	Q^2^
PSI	0.636	0.373
SI	0.203	0.153

## Conclusion

With the development of society, social media has increasingly become one of the main ways of communication. Under the influence of the COVID-19 crisis, short videos have been rapidly integrated into people’s daily life and have been used in all walks of life, including tourism. People’s sharing of tourism information has changed from blog graphics to short video sharing, which also adds a new way to disseminate tourism information and the publicity of tourism information. With the development of the times, the information communication of the tourism industry has kept up with the trend of the times to attract more tourists to pay attention to tourism information. This study takes tourism as the background and uses the theory of parasocial relationship. The results show that parasocial relationship is an important factor affecting people’s ability to share tourism videos. Emotional engagement, value congruence and entertainment motivation are important preconditions, which affect the parasocial relationship between tourists and tourism Vloggers. Parasocial relationship has a positive impact on sharing intention. Parasocial relationship and willingness to share play an intermediary role.

### Emotional engagement, entertainment motivation and value congruence to parasocial relationship

The results show that the emotional engagement of tourists and short video tourism Vloggers will positively impact the parasocial relationship of the audience. That is, when the audience have emotional factors with the short video tourism blogger, when the emotional components are added, the audience build a stronger sense of quasi-social interaction with the short video tourism blogger, which means more recognition or trust in the tourism information introduced by the tourism blogger or the recommended tourist attractions. Previously, some scholars have studied the relationship between emotional engagement and parasocial relationship. The results are consistent with the results of this study. Emotional engagement can positively affect parasocial relationship (Lim et al., 2020). Schramm and Wirth (2010) and Tsiotsou (2015) have shown that positive emotional outcomes contribute to the development of parasocial relationship. Emotional engagement has become the most important phenomenon to distinguish short video programs from watching TV programs on YouTube and other non-interactive online platforms. The present study mainly uses the social media platform to study the audience’s willingness to share short tourism videos. According to previous studies, emotion will affect people’s attitude and behavior, and emotional engagement is an important factor affecting parasocial relationship.

Consistent with the research results of Bi et al. (2021), tourists’ entertainment motivation has a positive impact on parasocial relationship. Bi et al. (2021) apply the concept of parasocial relationship (PSI) to TV programs to determine whether it will trigger the travel intention of young viewers. The results show that three of the four dimensions (entertainment, information and relaxation) predict the audience’s PSI and improve their perceived well-being and travel intention. Therefore, entertainment motivation will also affect the close relationship between tourists and tourism Vloggers to a great extent. Strong entertainment motivation will also improve the probability of interaction between tourists and Vloggers to increase their interest in tourism Vloggers.

Thus, establishing a strong parasocial relationship between the audience and the broadcaster is also accompanied by a closer relationship, improving the willingness to share. The value consistency of video tourism Vloggers has a positive impact on the parasocial relationship of the audience. Perhaps the previous research results of many scholars are consistent (Gong and Li, 2017; Haobin Ye et al., 2021; Jin et al., 2021). Shan et al. (2020) studied the common phenomenon of homogeneity in the context of dynamic social media in China, which is an important antecedent of parasocial relationship between audience and media roles (Rubin and Step, 2000; Tian and Hoffner, 2010; Lee and Watkins, 2016) Behavioral response shows that a celebrity with appearance consistency and value consistency to promote the brand, especially that value consistency is more influential than appearance consistency (Seomoon, 2019). In terms of tourism, in the research of tourism websites in communication media, Haobin Ye et al. (2021) discussed the antecedents and consequences of parasocial relationship between customers and social media spokesmen of tourism companies. Previous studies reveal that value consistency can positively impact social relations in different fields. From media spokesperson to tourism industry, value consistency and quasi-social interaction between tourists and short video tourism Vloggers is expanded.

### Emotional engagement, entertainment motivation and value congruence to sharing intention

Tourists’ parasocial relationship with the short video tourism blogger will affect tourists’ willingness to share the short video. When the parasocial relationship components are added, tourists’ intention to share the short video will be stronger, meaning they more agree with or believe in the tourism information introduced by the tourism blogger or the recommended tourist attractions. It may lead to a willingness to recommend the travel information transmitted by the short video of the travel blogger with friends or people in need. In previous studies, few scholars have explored the relationship between parasocial relationship and willingness to share. The credibility of influencers and parasocial relationship have a significant positive relationship with purchase intention (Sokolova and Kefi, 2020). Bi et al. (2021) applied the concept of parasocial relationship to TV programs to determine whether it will trigger the travel intention of young viewers. All the above results show that parasocial relationship has an impact on intention. This study finds that parasocial relationship has a positive impact on sharing intention, which is also in line with the same research results as previous scholars. They all belong to intention.

The impact of value congruence on parasocial relationship is also obvious. Cazier et al. (2007) agree with the results of this study that value consistency has a direct impact on people’s will. Values provide people with multiple functions and guide behavior and judgment in specific situations (Kahle and Lakes, 1983). To some extent, the consistency of values can determine whether people agree with the views of tourism Vloggers on the introduction of scenic spots. Tourists will share the information they think is good or useful. The study also found that values affect all aspects of consumer behavior. For example, the consistency of values significantly impacts brand attitude and purchase intention (Pradhan et al., 2016).

As for emotional participation and willingness to share, the results of this study show that emotional participation has no direct impact on willingness to share. The results of emotional engagement and willingness to share are contrary to those of Lata et al. (2021). The results show that the existence of emotion has a positive impact on willingness to share. This study shows that emotional engagement has no direct impact on sharing intention, possibly because of different research backgrounds. In the relationship between tourists and short video tourism Vloggers, a single emotion is not enough to become the reason for tourism information sharing. Many factors can cause sharing, such as the social status of the tourism blogger or the recognition of publishing information with the tourism blogger. Tamta and Rao (2017) shows a significant negative correlation between turnover intention and employees’ cognitive and emotional engagement. Although having a different research background from the present study, the study also confirms no direct impact between emotional engagement and willingness.

### The mediating role of parasocial relationship

Social interaction is of great significance as an intermediary variable between emotional engagement, value harmony and willingness to share. Compared with previous studies, the parasocial relationship between the audience and short video tourism Vloggers as the intermediary effect of emotional engagement and sharing intention has not been confirmed. Labrecque (2014) adopts the theory of parasocial relationship, which can be used as a theoretical perspective for designing successful social media strategies. The study used various methods to provide evidence of the role of PSI in developing positive relationship outcomes. Parasocial relationship has a full mediating effect on the impact of physical attractiveness on perceived brand and has a partial mediating effect on the impact of social attractiveness, entertainment motivation, relationship building motivation and time spent in the media on perceived brand quality (Liu et al., 2019). Parmar and Mann (2021) support that parasocial relationships mediate between celebrity image and purchase intention. This study takes short videos as an opportunity to study the relationship between short videos and tourism Vloggers’ willingness to share short videos. Parasocial relationship is an important theory. This study verifies the intermediary relationship of parasocial relationship. Parasocial relationship is an intermediary between tourists’ and short video tourism Vloggers’ emotional engagement, value congruence and entertainment motivation. The results show that parasocial relationship also has mediating effect in the new disguised relationship.

## Implications

### Theoretical implications

This study has certain significance contributions to marketing theory. Firstly, although the photographers of short videos have been affected, some scholars have paid attention to short videos but discussed them in market media. However, the role of tourism-related short video Vloggers in parasocial relationships has not been discussed in tourism literature. From the perspective of social psychology, most people only stay on the surface of the relationship between the short video influencer and its tourists and lack an in-depth understanding of the relationship between the short video influencer and its tourists. Taking short videos as the background, this study discusses the important relationship between similarity attraction theory and parasocial relationship theory. This study contributes to future research to identify specific aspects of values related to social interaction.

Secondly, this study complements the study of parasocial relationship theory by exploring the relationship between tourists’ values, entertainment motivation and emotional participation. In the field of media research, current research shows that two factors lead to parasocial relationship. Social and physical interaction are important factors affecting parasocial relationship (Kurtin et al., 2018). The study added that personality and values also produce parasocial relationships. We have extended the study of parasocial relationships by introducing theories of parasocial relationships based on websites or blogs into the field of short video tourism.

Third, most research on parasocial relationships has focused on their direct impact. We extend the literature by exploring the mediating influence of parasocial relationships between emotional engagement, value congruence, entertainment motivation, and online sharing intentions. The findings suggest that the more substantial the parasocial relationship, the greater the likelihood of sharing short videos. Specifically, the stronger the parasocial relationship between tourists and travel Vloggers, the more likely they were to share travel information.

Finally, most studies on parasocial relationship and its consequences are based on purchase impact. This study takes tourism short video Vloggers as the research object to explore the impact of emotional engagement on the relationship between parasocial intention and sharing intention. The results show that parasocial intention has a direct effect on sharing intention. This study expands the existing literature on quasi-social interaction by revealing the antecedents and consequences of quasi-social interaction in tourism short video sharing intention. Previous studies mainly focused on websites and spokespersons (Gong and Li, 2017), but no study has been conducted on the correlation between quasi-social interaction based on tourism short videos and willingness to share. This study adds the theory of sharing intention, extends previous studies, and finds that parasocial relationships strongly impact sharing willingness in a new field. This study proves the instrumental utility of quasi-social interaction, which is helpful to the research in this field.

### Practical implications

The study results are conducive to the sustainable development of the tourism industry and provide rich management inspiration for the development of tourism marketing after the COVID-19 pandemic. The current study will provide some development suggestions for the online publicity and sharing of tourism or scenic spots. According to our research results, we can make the following suggestions. Firstly, based on the conclusion that emotional engagement, entertainment motivation and value congruence will lead to more interactive behavior of the tourists, we suggest that scenic spots can enhance their popularity by strengthening audience interaction. For example, they can use short videos of tourism experts to promote scenic spots. Tourists can be invited to scenic spots to shoot short videos in the same scenic spot. Tourists can receive a lot of relevant information about scenic spots through the publicity of short videos and will also open it in the same place with tourism Vloggers to improve the popularity of the scenic spot.

Secondly, the research shows that parasocial relationships will enhance the tourists’ willingness to share. In previous studies, the attractiveness of media roles will lead to more tourist parasocial relationships (Rosaen and Dibble, 2008). Therefore, strengthening the social relationship of the tourists is more important for the parasocial relationship on the scene. We can start from this aspect to develop the publicity and marketing of scenic spots. For example, when publishing short videos of scenic spot related information, you can use various functions of the short video platform, including direct message, comment, post, reply, like and lottery, so that tourists can experience closer parasocial interaction and feel a stronger sense of social existence. Tourist destinations can also invite short video tourism Vloggers to publicize some leisure tourism scenic spots around the tourist destinations, develop near outing tourism projects, attract some nearby tourists to travel and improve the tourism industry after the outbreak of the new pavilion epidemic.

Finally, emotional participation has a particular impact on social interaction. Scenic spots can improve quasi-social interaction by improving tourists’ emotional participation. For example, under short videos, the audience’s questions can be answered in the comment area under short videos, and some topics of interest can be said. Some tourism Vloggers can be invited to do some offline tourism sharing activities to improve the emotional communication between tourists and short video tourism anchors. Short video tourism Vloggers can also count the tourism knowledge they want to know most through the comment area, select several as representatives and shoot video for answers. Thus, the emotional communication between tourists and short video tourism Vloggers is increased, more parasocial relationships are promoted, and more sharing intention is increased. The above suggestions will help to improve the popularity of scenic spots or tourist cities and restore the tourism economy. Suggestions for the diversified development of tourism and the sustainable development of the tourism industry after the epidemic fluctuation are provided.

## Conclusion, limitations and future research

Developing short video tourism is an important measure for the sustainable development of the network economy. Since the COVID-19 outbreak, tourism has witnessed the surge of social media in promoting the relationship between scenic spots and visitors. The potential mechanism of how social media can promote the construction of the relationship between scenic spots and audience has not been revealed. This study integrates parasocial relationship theory and sharing intention theory. The purpose is to explain how short video tourism Vloggers affect the relationship between online users’ willingness to watch and share to narrow the gap in this research field. The results show that the similarity in entertainment motivation, emotional engagement and values between short video tourism Vloggers and tourists leads to the parasocial relationship between them, which affects their sharing intention. This study enriches the research theory in social media short video and provides reference suggestions for online sales of the scenic spot industry. Although the conclusion of this study has a particular contribution to the existing theory, it provides some practical enlightenment for the online development of tourism destinations.

However, this study inevitably has many research limitations in research scope, research methods, research design and research depth; it also provides space and theoretical ideas for follow-up research, mainly including the following aspects.

Firstly, the research sample of this study has certain limitations. Internet economy will be different in different periods and countries. Therefore, in future research, samples can be collected in different countries and at different times to verify the applicability of the research model in different contexts.

Second, there are certain limitations in research methods, quantitative research can well deal with the “relationship, interaction and causal relationship between variables,” but it cannot pay more attention to the relationship between phenomenon and background, the process of phenomenon change and the significance of phenomenon and behavior to behavior subjects as qualitative research.

Finally, the cross-sectional sampling method used in this study, which may affect the estimation of hypothesis relationships.

These limitations should be considered when interpreting the results of the current study. In the future, encourage more research on social media short video tourism Vloggers’ antecedents in parasocial relationships. Future research can also compare different social media celebrities, which may produce profound findings aimed at parasocial relationships. Some boundary conditions should also be revealed, which may change the intensity of parasocial relationships.

## Data availability statement

The original contributions presented in the study are included in the article/supplementary material, further inquiries can be directed to the corresponding author.

## Ethics statement

Ethical review and approval were not required for the study on human participants in accordance with the local legislation and institutional requirements. Written informed consent from the participants was not required to participate in this study in accordance with the national legislation and the institutional requirements.

## Author contributions

All authors listed have made a substantial, direct, and intellectual contribution to the work and approved it for publication.

## Conflict of interest

The authors declare that the research was conducted in the absence of any commercial or financial relationships that could be construed as a potential conflict of interest.

## Publisher’s note

All claims expressed in this article are solely those of the authors and do not necessarily represent those of their affiliated organizations, or those of the publisher, the editors and the reviewers. Any product that may be evaluated in this article, or claim that may be made by its manufacturer, is not guaranteed or endorsed by the publisher.

## Footnotes

^1^ www.wjx.cn
